# Effect of Geography on the Analysis of Coccidioidomycosis-Associated Deaths, United States

**DOI:** 10.3201/eid2210.160696

**Published:** 2016-10

**Authors:** Jason A. Noble, Robert G. Nelson, Gudeta D. Fufaa, Paul Kang, Shira Chani Shafir, John N. Galgiani

**Affiliations:** University of Arizona College of Medicine, Phoenix, Arizona, USA (J.A. Noble, P. Kang);; National Institutes of Health, Bethesda, Maryland, USA (R.G. Nelson, G.D. Fufaa);; University of California, Los Angeles, California, USA (S.C. Shafir);; University of Arizona College of Medicine, Tucson, Arizona, USA (J.N. Galgiani)

**Keywords:** Coccidioides, coccidioidomycosis, diabetes mellitus, endemic diseases, African Americans, Arizona, California, Hispanic Americans, Native Americans, North America, risk factors, southwestern United States, spores, fungi

## Abstract

Because coccidioidomycosis death rates vary by region, we reanalyzed coccidioidomycosis-associated mortality in the United States by race/ethnicity, then limited analysis to Arizona and California. Coccidioidomycosis-associated deaths were shown to increase among African-Americans but decrease among Native Americans and Hispanics. Separately, in a Native American cohort, diabetes co-varied with coccidioidomycosis-associated death.

In a recent study (*1*), researchers used a publically available database derived from death certificates to identify factors associated with deaths attributed to coccidioidomycosis in the United States. However, because coccidioidomycosis is endemic to only a few states, especially Arizona and California, and the racial/ethnic compositions of these states do not reflect the country as a whole, we refined the original analysis to compare national statistics with those of Arizona and California. Here we report the differences observed after using relevant demographic variables that align with the epidemiology of serious coccidioidal infections.

## The Study

We analyzed multiple-cause-of-death data by using established methods (*1*). We then restricted analysis to Arizona and California. Publically available data from the Centers for Disease Control and Prevention National Center for Health Statistics (https://www.cdc.gov/nchs) for 1990–2008 were used to calculate national and state-specific mortality rates. We included in our analysis all coccidioidomycosis-associated deaths (indicated by codes 114.0–114.9 from the International Classification of Diseases (ICD), Ninth Revision, or B38.0–B38.9 from the ICD, Tenth Revision) and calculated mortality rates by using bridged-race population estimates from US census data. We age-adjusted these rates by using weights from the 2000 US census and then calculated incidence rate ratios accordingly. We used a generalized linear model with a log-binomial construct to analyze the effect of sex, age, and race/ethnicity. Interaction terms between race/ethnicity and sex were not statistically significant (United States overall 0.96 [p = 0.15], Arizona 0.92 [p = 0.11], California 0.96 [p = 0.37]).

Our analysis of national coccidioidomycosis-associated deaths confirmed what was previously reported (*1*) (Table). During 1990–2008, a total of 3,088 coccidioidomycosis-related deaths were reported in the United States. Men were disproportionately affected. Although most decedents were non-Hispanic whites, the mortality rate among non-Hispanic whites was the lowest among all racial/ethnic groups reported. For other groups, coccidioidomycosis-related deaths were highest among Hispanics and Native Americans and lowest among African-Americans and Asians.

**Table:** Differences in coccidioidomycosis-associated deaths, by geography, sex, and race/ethnicity, Arizona, California, and United States overall, 1990–2008

Characteristic	US mortality rate*	Incidence rate ratio† (95% CI)				Relative risk‡ (95% CI)		
		United States	Arizona	California		United States	Arizona	California
Sex								
F	0.31	Referent	Referent	Referent		Referent	Referent	Referent
M	0.93	3.03 (2.80–3.27)	2.24 (1.95–2.54)	3.49 (2.68–3.69)		2.32 (1.97–2.73)	1.76 (1.38–2.26)	2.79 (2.10–3.71)
Race/ethnicity								
Non-Hispanic white	0.40	Referent	Referent	Referent		Referent	Referent	Referent
Hispanic	1.82	4.45 (4.06–4.88)	1.36 (1.13–1.64)	2.48 (2.09–2.96)		6.74 (5.59–8.14)	2.10 (1.53–2.88)	5.21 (3.87–6.99)
African American	0.69	1.70 (1.52–1.91)	3.41 (2.65–4.38)	5.15 (4.27–6.22)		1.38 (1.16–1.60)	3.42 (2.55–4.60)	3.50 (2.84–4.31)
Asian	1.25	2.84 (2.42–3.35)	2.19 (1.38–3.49)	1.88 (1.51–2.37)		5.48 (4.32–6.79)	5.51 (2.82–10.7)	2.70 (2.01–3.61)
Native American	2.67	6.52 (5.14–8.30)	2.50 (1.91–3.28)	1.39 (0.58–3.38)		8.15 (6.09–10.9)	2.18 (1.52–3.12)	3.01 (1.31–6.89)

*Per 1 million person-years. †Univariate analysis (sex or race/ethnicity). ‡Multivariate analysis (sex and race/ethnicity).

After we restricted analyses to Arizona and California, where coccidioidomycosis is endemic, coccidioidomycosis-related mortality rates were 2.19 (95% CI 1.38–3.49) and 1.89 (95% CI 1.51–2.37) deaths per 100,000 person-years, respectively. A total of 1,957 coccidioidomycosis-related deaths were reported in Arizona and California during 1990–2008. In both states, mortality rates were highest among men, and compared with non-Hispanic whites, all other racial/ethnic groups had higher mortality rates attributed to coccidioidomycosis. However, in contrast with the national data, African-Americans had the highest and Native Americans and Hispanics the lowest excess mortality compared with non-Hispanic whites. Risk for coccidioidal death increased with age (Figure). Moreover, death rates attributed to coccidioidomycosis were higher across virtually all age groups in the highly endemic states, especially in Arizona, than in the United States as a whole. The only exception to this finding was that there were no deaths among persons <15 years of age in California. A multivariate analysis indicated that older age, male sex, and nonwhite race were independent risk factors for coccidioidomycosis-associated mortality.

**Figure:**
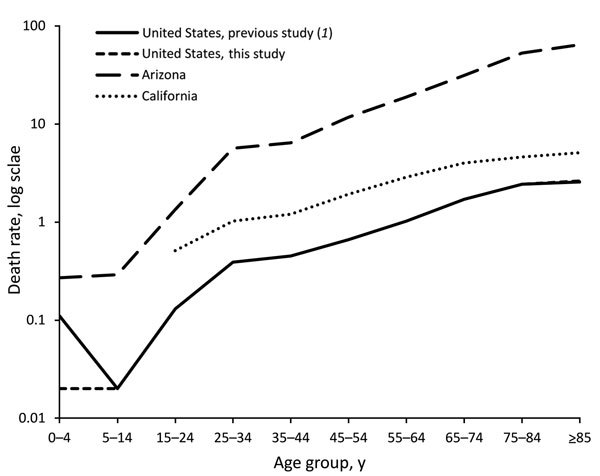
Coccidioidomycosis-associated mortality rates, by age group, Arizona, California, and United States overall, 1990–2008. The difference in the mortality rate of the 0–4 year age group between previous study (*1*) and this study is attributable to a misprint in the source document.

Separately, we examined the coccidioidomycosis-related deaths in a Native American population residing near Phoenix, Arizona. We consulted a longitudinal study of diabetes and its complications within the Gila River Indian Community (GRIC) conducted during 1965–2007, a subset of which is published (*2*). The results of that study cover a well-characterized population of persons who are of >50% Pima Indian heritage. We included in our analysis all persons >15 years of age who resided within the community. We considered all deaths with codes 114.0–114.9 from the ICD, Ninth Revision, listed as the underlying or contributing cause of death to be coccidioidomycosis-associated deaths. We calculated mortality statistics within the GRIC by using population estimates from the 1980 GRIC census and then adjusted these estimates for age and sex.

During 1965–2008, a total of 17 coccidioidomycosis-related deaths were reported in the GRIC. In this cohort, there was no male predominance (9 of the decedents were women). All deaths occurred in persons >45 years of age (range 49–86 years). Death in this group was highly associated with diabetes (15 of the decedents had type 2 diabetes). The overall crude mortality rate was 350 deaths/1 million person-years, and the age-sex adjusted mortality rate was 6.3-fold (95% CI 1.8–11.6-fold) higher among those with diabetes compared with those without.

## Conclusions

Although the effect of race/ethnicity on dissemination of coccidioidomycosis has been reported previously (*3*–*6*), little is known about race/ethnicity and coccidioidomycosis-attributed death. By using equivalent methods, we confirmed many of the findings reported by Huang et al. (*1*). We then wondered how a reanalysis, using only the populations at the highest risk for exposure to *Coccidioides* spores, would change the associated mortality incidence rate ratios and relative risks.

In 2000, Arizona had a population of ≈5 million. With ≈80% of the population residing within the highly coccidioidomycosis-endemic area of Maricopa and Pima counties, ≈4.1 million persons were at risk for exposure to *Coccidioides*. In 2000, California had a population of ≈34 million. Only 4% of the population (≈1.5 million) lived in the counties comprising the San Joaquin Valley. These 5.6 million persons, who are most likely to inhale *Coccidioides* spores, make up only ≈2% of the US population (*7*,*8*). With this in mind, we considered how the mortality rate might change if we restricted analysis to the 2 states with the highest endemicity.

Restricted analysis confirmed that the mortality rate increased with age and was associated with male sex. These associations are in agreement with previous reports (*9*,*10*). Increased mortality was observed in nonwhite racial/ethnic groups. However, in contrast to the national data, African-Americans had the highest coccidioidomycosis-associated mortality rate, whereas risk for Hispanics, Native Americans, and Asians was less elevated compared with non-Hispanic whites. These findings correspond well with what has been reported by Seitz et al. (*6*), that nonwhite race is a risk factor for disseminated coccidioidomycosis-associated hospitalization in Arizona and California.

Examination of the GRIC allowed us to calculate coccidioidomycosis-related mortality rates in a genetically well-defined population. The high coccidioidomycosis-related mortality rate in Pima Indians is probably related at least in part to the high incidence of comorbidities in this population. Further, because none of these deaths occurred in persons <45 years old, the increased mortality rate among Native Americans is probably not solely related to genetic susceptibility; however, persons <15 years of age were not included in the GRIC analysis, which might limit this interpretation. Instead, the increased mortality rate is more probably a result of the high rate of debilitating comorbidities in this population. This argument is supported by the longer duration of diabetes associated with increased infection-related mortality in Pima Indians (*11*).

Our study highlights the advantage of calculating rates of endemic diseases within their respective regions. By confining analysis to Arizona and California, our study measures the race/ethnicity-associated risk for death attributed to coccidioidomycosis in a highly disease-endemic region, which differs from the United States overall. Mortality rates are higher for men and all nonwhite racial/ethnic groups, especially for African-Americans. Sex and race/ethnicity affected mortality rates independently. Whether this predilection for nonwhite groups is attributable to genetic susceptibility, socioeconomics, or other factors remains to be determined. Data from a Native American population suggest a relationship between diabetes and coccidioidomycosis-associated death. Whether this phenomenon is applicable to other populations deserves further investigation. We caution against strict interpretation of these data because multiple-cause-of-death data are inherently limited by their use of population estimates and death certificates for statistical analysis (*12*).
